# Sympathetic ophthalmia: to the twenty-first century and beyond

**DOI:** 10.1186/1869-5760-3-49

**Published:** 2013-06-01

**Authors:** Xi K Chu, Chi-Chao Chan

**Affiliations:** 1 Immunopathology Section, Laboratory of Immunology, National Eye Institute, National Institutes of Health, 10 Center Drive, Room 10N103, Bethesda, MD 20892, USA

**Keywords:** Sympathetic ophthalmia, Dalen-Fuchs nodules, Inflammation, Enucleation, Corticosteroids, Ocular imaging

## Abstract

Sympathetic ophthalmia is a rare bilateral granulomatous inflammation that follows accidental or surgical insult to the uvea of one eye. Onset of sympathetic ophthalmia can be insidious or acute, with recurrent periods of exacerbation. Clinical presentation shows mutton-fat keratic precipitates, choroidal infiltrations, and Dalen-Fuchs nodules. Histopathology reveals diffuse or nodular granulomatous inflammation of the uvea. Prevention and treatment strategies for sympathetic ophthalmia are currently limited to two modalities, enucleation of the injured eye and immunosuppressive therapy, aimed at controlling inflammation. The etiology and pathophysiology of the disease is still unclear but is largely thought to be autoimmune in nature. Recent insight on the molecular pathology of the disease as well as developments in imaging technology have furthered both the understanding on the autoimmune process in sympathetic ophthalmia and the targeting of prevention and treatment strategies for the future.

## Review

### Introduction

Despite the long history of sympathetic ophthalmia, much is still to be elucidated about the pathophysiology of the rare, bilateral, non-necrotizing granulomatous uveitis [1]. Sympathetic ophthalmia presents with posterior inflammation that may include optic nerve swelling, exudative retinal detachment, and anterior granulomatous inflammation with mutton-fat keratic precipitates in severe and/or chronic recurrent cases [1,2]. The rarity of the disease has rendered study on its incidence difficult, and estimates from reported series have yet to establish any general consensus, although sympathetic ophthalmia appears to have no predilection toward any particular age, race, or gender [3]. That the injured eye, known as the *exciting eye*, and the contralateral eye, or *sympathizing eye*, demonstrate similar pathology suggests involvement of an autoimmune response. Corticosteroid therapy that targets inflammation systemically is considered a mainstay of treatment after onset of sympathetic ophthalmia [4,5].

### Sympathetic ophthalmia to the twenty-first century

#### History

Noted by Hippocrates more than two millennia ago and intermittently cited in the seventeenth and eighteenth centuries through clinical report, the disease that places ‘the other eye in great danger’ following ocular injury has a long history but was not fully defined until the turn of the nineteenth century [3]. William Mackenzie provided the first full clinical description of the disease, coining the term ‘sympathetic ophthalmitis’ in 1840 [6].

While Mackenzie's was the first clinical description of sympathetic ophthalmia in full, the complete histopathological report of the disease did not appear until 1905, with a publication by Ernst Fuchs describing infiltration of the uveal tract, particularly the choroid, and formation of nodular aggregations beneath the retinal pigment epithelium (RPE) in several specimens [3]. These nodules, termed Dalen-Fuchs nodules, had been noted previously by his contemporary, Dalen [3]. Fuchs and many others noted that excluding trauma, the pathology of the sympathizing eye was similar to that of the exciting eye. So definitive was Fuchs' work that even half a century later, little more had been added to the knowledge on histopathology of sympathetic ophthalmia.

#### Epidemiology

The incidence of sympathetic ophthalmia is contentious as the disease is agreed to be rare, but the exact occurrence remains disputed. While literature on the incidence of sympathetic ophthalmia is available, the insufficient number of case series available for study has created difficulty in establishing a conclusive value. More recent studies reported incidence to range from 0.2% to 0.5% following injury and 0.01% following intraocular surgery [7,8]. Increased knowledge on sympathetic ophthalmia and other ocular inflammatory diseases may have sharpened diagnostic ability and, to some degree, may account for the lower incidence levels reported in more recent literature.

The disease appears to have no predilection towards any particular sex, race, or age, although some studies have found a higher incidence of sympathetic ophthalmia in males; one survey at the Massachusetts Eye & Ear Infirmary attributed two-thirds of cases to males and one-third to females [3]. It is suggested, however, that this is accounted for by the underlying increased exposure of males to injury than that of their female counterparts. With regard to age, sympathetic ophthalmia follows normal distribution with some report of higher occurrence among children and individuals over 60 years of age, likely arising from increased ocular injury in young age and increased frequency of intraocular surgery in the older population [3].

#### Clinical features and differential diagnosis

**Figure 1:**
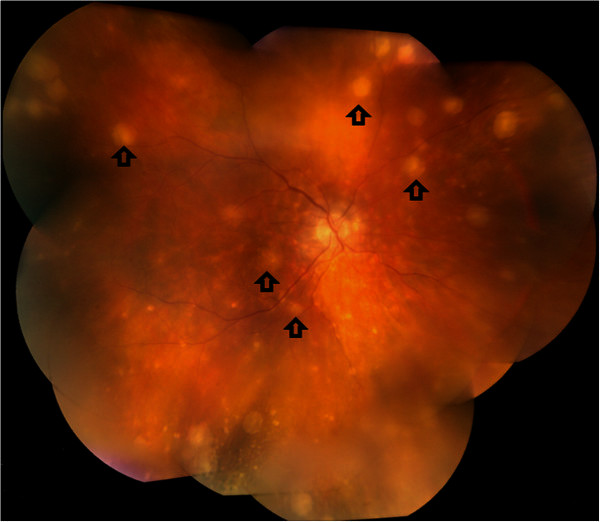
**Montage fundus photograph of a sympathetic ophthalmia retina.** Yellow-white subretinal spots (arrows) on clinical fundus photographs correspond to histopathological Dalen-Fuchs nodules and choroidal infiltrates.

The onset of sympathetic ophthalmia is variable, appearing anytime between 1 week and 66 years after the inciting injury [9], with a reported majority of cases (90%) occurring within a 1-year time span [10]. Patients with sympathetic ophthalmia classically present with bilateral anterior uveitis associated with mutton-fat keratic precipitates and moderate to severe vitritis, choroiditis, and papilitis in the posterior segment [11]. It is described that at initial onset, the main clinical findings such as optic nerve swelling and exudative retinal detachment are located in the posterior segment, while granulomatous anterior segment inflammation with mutton-fat keratic precipitates may be seen in severe and/or chronic recurrent cases [12-14]. Sub-RPE nodular lesions that appear yellow-white, corresponding to histopathologic Dalen-Fuchs nodules (Figure 1), are typical of sympathetic ophthalmia [2].

While these nodules were initially characterized in sympathetic ophthalmia cases, they are not pathognomonic [15] and may appear only in approximately one-third of enucleated eyes with sympathetic ophthalmia [16,17]. Complications of sympathetic ophthalmia include secondary cataract, glaucoma, and chronic maculopathy [18]. Patients with sympathetic ophthalmia can experience recurrent episodes of exacerbation and, on rare occasion, extra-ocular symptoms such as hearing loss, headache, vitiligo, and meningeal irritation [19].

Differential diagnosis of sympathetic ophthalmia is made primarily on patient history and clinical presentation, with approximately 20% of cases being confirmed through histology [20]. While it is generally agreed that the inciting event behind sympathetic ophthalmia is penetrating injury, some exceptions such as non-perforating ocular procedures and laser therapies have been associated with the disease [21]. Other causes of granulomatous uveitis must also be dismissed before a diagnosis of sympathetic ophthalmia can be made, particularly Vogt-Koyanagi-Harada syndrome (VKH) and sarcoidosis, both of which have systemic involvement and absence of ocular injury [18].

#### Histopathology and immunopathology

The general finding in sympathetic ophthalmia is uveal granulomatous inflammation primarily by lymphocytes, surrounding macrophages, and some multinucleated giant cells. The inflammation is comprised of T lymphocytes that switch from a predominant composition of CD4+ helper T cells in the early stage of the disease to a later predominance of CD8+ cytotoxic T cells [22]. B cells are identified in less than 5% to 15% of choroidal infiltrate [23]. It is classically described that the retina and choriocapillaries are spared in the inflammatory process [24]. Other studies, however, have implicated the choriocapillaries in disease progression, with one study reporting 40% of sympathetic ophthalmia patients with choriocapillary involvement and another noting chorioretinal scarring in 25% of cases [11].

**Figure 2:**
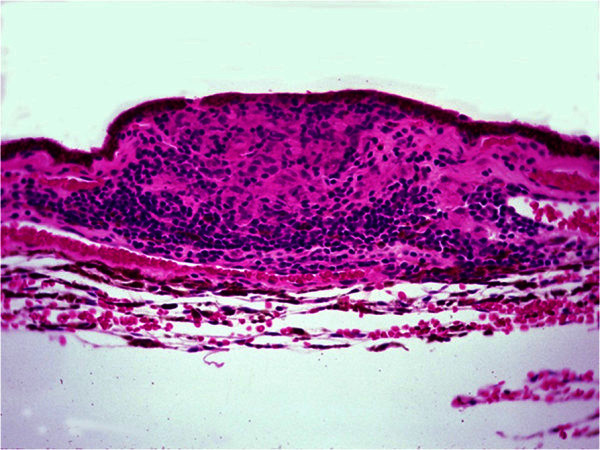
**Histopathology of a Dalen-Fuchs nodule.** There is an accumulation of mainly macrophages beneath the retinal pigment epithelium (brown color). Hematoxylin & eosin; original magnification, ×200.

Dalen-Fuchs nodules are a well-known feature of sympathetic ophthalmia that appears in 25% to 35% of cases [17]. They are primarily located in the choroid beneath the RPE or under the neuroretina. Dalen-Fuchs nodules undergo development, in which they are primarily composed of macrophages early on (Figure 2) but later may be composed of depigmented or degenerated RPE and a small number of lymphocytes [25,26].

#### Etiology

The original hypothesis proposed by Mackenzie and his contemporaries in the nineteenth century suggested the bilateral impact of sympathetic ophthalmia to be the result of inflammation in the injured eye that propagated along the optic nerve and chiasma to the contralateral eye. In the early twentieth century, it was proposed that the disease was a hypersensitivity reaction and that the antigen responsible was melanin [27]. The strong correlation between uveal injury and sympathetic ophthalmia also led some to suspect the pathogenic role of the uvea, citing the presence of antiuveal antibodies in a large percentage of patients with sympathetic ophthalmia.

The most convincing theory thus far points to the role of cell-mediated immune response to antigens from the retinal photoreceptor layer. While the particular antigen is yet to be determined, putative retinal antigens include retinal soluble antigen (S-antigen) [28], rhodopsin [29], interphotoreceptor retinoid-binding protein [30], and recoverin [31]. Retinal S-antigen has been the most extensively studied, and although cell-mediated immune response to S-antigen has been demonstrated in animal models, circulating S-antigen antibody has not been detected in the sera of all patients with sympathetic ophthalmia [32]. Melanin antigens have also been shown to induce uveitis wherein CD4+ T helper cells are mediators of inflammation, and the primary target tissue is the uvea. This model, experimental melanin protein-induced uveitis, rarely involves the retina and instead shows inflammation most evident in the iris, ciliary body, and choroid [33,34], pathological features that are spatially more similar to that of sympathetic ophthalmia.

Lymphatics and their role in a mechanism of sensitization have also been implicated in sympathetic ophthalmia [32]. In the intact eye, the intraocular antigens bypass local lymph nodes and circulate directly to the blood and spleen. Following injury, however, uveal tissue is exposed to conjunctival lymphatics, and antigens move to the regional lymph nodes, resulting in a cell-mediated immune response. Rao et al. showed induction of uveitis through injection of retinal antigens with adjuvant under the conjunctiva but not into the eye [35]. That the concurrent presence of an infectious agent with an antigen is necessary to incite immune response, to serve as adjuvants in this mechanism of sensitization, has been suggested.

Genetic predisposition to the development of sympathetic ophthalmia has also been proposed, with particular focus on human leukocyte antigen (HLA). HLA-A11 antigen expression has been linked to patients with sympathetic ophthalmia, as well as HLA-DRB1*04, DQA1*03, and DQB1*04 [36,37]. Interestingly, similar associations have been recognized between HLA-D4, DQw3, and -DRw43 and VKH disease [36].

#### Prevention and therapy

Management for sympathetic ophthalmia has been classically divided into two modalities: enucleation for prevention and corticosteroid therapy for treatment. First practiced in 1851 by Pritchard, enucleation is generally recommended within 2 weeks post-injury [3,38]. Considerable controversy has existed around both promptness of action, as sympathetic ophthalmia has been reported as early as 5 days after injury, and appropriateness of the procedure, as the exciting eye may actually present with better vision than the sympathizing eye in the course of the disease. Some have suggested early enucleation of the injured eye to improve visual prognosis of the sympathizing eye [17], while others note that reviews of sympathetic ophthalmia cases from a histology standpoint show no benefit from enucleation of the exciting eye [39].

Corticosteroids have served as the mainstay of treatment following onset [4]. High doses of oral corticosteroids are recommended for 3 months [40], upon which re-evaluation is needed. If inflammation has improved, corticosteroid therapy can be tapered in a period of 3 to 6 months. In cases in which patients have issues with corticosteroid tolerance or lack of response, other immunomodulatory agents such as cyclosporine and methotrexate can be used; however, these therapies should be closely monitored for high adverse effects [18,41].

### Sympathetic ophthalmia into the twenty-first century and beyond

#### Emerging theories of immune involvement

Growing evidence points to the role of autoimmunity in sympathetic ophthalmia, yet exact mechanisms are still to be defined. Recent findings put forth several promising theories that lend to a better understanding on the pathogenesis behind this disease. A 2007 study proposes a pathogenic role of leukocyte recruitment in sympathetic ophthalmia. Specifically, Abu El-Asrar et al. cite elevated gelatinase B (matrix metalloproteinase-9) and chemokines monocyte chemotactic protein-1 (CCL2/MCP-1) and stromal cell-derived factor-1 (CXCL12/SDF-1) within cells in granulomas of sympathetic ophthalmia [42]. Matrix metalloproteinases (MMPs), particularly gelatinase B (MMP-9), play an integral role in leukocyte migration and have been implicated with other chronic inflammatory and autoimmune diseases such as rheumatoid arthritis [43]. It is suggested that excessive cleavage by gelatinase B may produce immunodominant epitopes that are processed and presented on major histocompatibility complex class II molecules on the surface of antigen-presenting cells to result in the activation of autoreactive T cells. CCL2 upregulation in sympathetic ophthalmia is consistent with previous studies finding CCL2 immunoreactivity in macrophages, epitheliod cells, and multinucleated giant cells within granulomas, and *in vitro*, CCL2 is reported to be a potent chemotactic factor and regulator of cytokine production by monocytes [44]. In addition, a predominance of B cells in uveal infiltrate has been reported, contrary to previously reported T cell predominance but conceivably suggestive of B cell predominance in the final stages of the disease, implicating a possible B cell activation of autoreactive T cells [42]. Consistent with this result, elevated CXCL12 as discovered in this study points to pathogenesis in the recruitment of B lymphocytes, as previous reports suggest CXCL12 to be a critical chemoattractant in trafficking and migration of mature autoreactive B cells [45].

Suggestive cytokine and chemokine involvement in sympathetic ophthalmia pathogenesis has led to recent characterization and functional investigation into the cytokine milieu of the disease. In a recent study, Furusato et al. demonstrated elevated CXCL11, CCL19, IL-18, and IL-23, as well as high IL-17, in granulomatous infiltrates of sympathetic ophthalmia, and increased IFN-γ and CCL17 levels in non-granulomatous inflammatory infiltrates [46]. Importantly, these cytokine and chemokine expression profiles establish the predominance of M1 macrophages within granulomas and Dalen-Fuchs nodules and the key presence of Th1 cells in non-granulomatous infiltrates, providing a framework to understand the contribution of specific inflammatory cell subsets to the pathogenesis of sympathetic ophthalmia. Interestingly, a 2012 case report of an HIV-positive patient on highly active antiretroviral therapy (HAART) diagnosed with sympathetic ophthalmia 9 years after penetrating injury lends further evidence to previously reported CD4+ T cell involvement in the early disease stage. The report suggests that HIV-mediated CD4+ T cell deficiency impairs inflammatory response, halting development of sympathetic ophthalmia, an effect that is reversed by antiretroviral therapy. Up to 25% of HIV patients experience inflammatory syndromes as a result of T cell reconstitution following HAART therapy [47], and the authors of this case cite similar pathogenesis between CD4 count restoration in HIV and onset of sympathetic ophthalmia despite the absence of an identified infectious antigen in the latter disease [48].

In addition to the study on immune and inflammatory response, attention has recently been drawn to the potential role of photoreceptor oxidative stress in sympathetic ophthalmia. The loss of vision in the absence of inflammatory infiltrate in the retina and choriocapillaries as presented in sympathetic ophthalmia has long been an unanswered question in this disease. Observation of upregulated TNF-α in the early experimental autoimmune uveoretinitis (EAU) animal model for human autoimmune uveitis, before inflammatory cell infiltration of the retina, suggests a potential mechanism for vision loss in sympathetic ophthalmia. TNF-α and its receptor, inducible nitric oxide synthase (iNOS), as well as iNOS- and peroxynitrite (ONOO^−^)-induced oxidative stress products were found to immunolocalize to the inner segments of photoreceptors in sympathetic ophthalmia patients; in particular, iNOS and nitrotyrosine, an ONOO^−^-induced oxidative stress product and marker for ONOO^−^, localized to photoreceptor mitochondria. Upregulation of TNF-α has been reported to increase production of ONOO^−^, resulting in photoreceptor mitochondrial oxidative stress and photoreceptor apoptosis [49]. Based on these findings, Parikh et al. propose TNF-α-induced iNOS and formation of ONOO^−^, resulting in the nitration of photoreceptor mitochondria-related proteins such as cytochrome C, triggering the apoptosis cascade and leading to photoreceptor cell death [50].

Another study in early EAU reports nitration of photoreceptor mitochondrial proteins prior to macrophage infiltration, suggesting an initiating role of oxidative stress in the development of uveitis. Oxidative stress was suggested as an effect of a low level of retinal T cell infiltrates that caused production of cytokines, including TNF-α, that resulted in oxidative stress and subsequent recruitment of inflammatory cells [51]. Sympathetic ophthalmia may follow a similar mechanism.

The discovery of gene regulation by microRNA has helped shed further light on the mechanisms behind sympathetic ophthalmia pathogenesis. Human genome-wide microRNA array revealed downregulation of four microRNAs (hsa-miR-1, hsa-let-7e, hsa-miR-9, hsa-miR-182) associated with the inflammatory signaling pathway, and that of these four, one microRNA (hsa-miR-9) has validated targets associated with mitochondrial oxidative stress that are thought to result in photoreceptor cell death in sympathetic ophthalmia, TNF-α and NF-κB [52]. Downregulation of these microRNAs, in particular hsa-miR-9, may prompt inflammatory signaling and induce mitochondrial oxidative stress, resulting in photoreceptor cell death and consequential vision loss in sympathetic ophthalmia [52].

In a 2012 study, Kase et al. demonstrated αA-crystallin expression in the normal retina and αA-crystallin upregulation in the inner segments and outer segments of photoreceptors in the sympathetic ophthalmia eye [53]. Sparing of the retina is observed in sympathetic ophthalmia, suggesting a protective function of αA-crystallin against apoptosis in the photoreceptors of sympathetic ophthalmia eyes. αA-crystallin is demonstrated to have an anti-apoptotic function through the blockage of pro-apoptotic mitochondrial pathways, inhibiting downstream events [54]. That oxidative stress upregulates αA-crystallin and oxidative stress markers localize to the inner segments suggest that in sympathetic ophthalmia eyes, increased αA-crystallin expression may be the result of photoreceptor oxidative stress in this disease [53].

#### Changing epidemiology

While the prevalence of sympathetic ophthalmia is widely cited to be 0.03 out of 100,000 per year [55], developments in the contributing factors behind sympathetic ophthalmia have led to changing prevalence of the disease. A decrease in military engagement would suggest falling rates of sympathetic ophthalmia as the probability of ocular injury from battlefield trauma decreases. Caution must be taken, however, against dismissive attitudes toward potential stagnancy or upward incidence of the disease.

The increasing prevalence of ocular surgery may play a role in the epidemiology of sympathetic ophthalmia. Earlier studies reported sympathetic ophthalmia following trauma to be most likely; however, more recent studies report injury from ocular surgery to be the more common cause [55]. Although incidence of sympathetic ophthalmia has declined in the recent past, prevalence of ocular surgery is increasing and necessitates careful monitoring of changing disease incidence. Sympathetic ophthalmia is a concern following multiple intraocular surgeries such as cataract extraction, paracentesis, and iridectomy, and is of particular concern following vitreoretinal surgery. Vitreoretinal surgeries such as pars plana vitrectomy and retinal detachment repair are risk factors for sympathetic ophthalmia, either through trauma-related issues or rhegmatogenous retinal detachment [56]. To date, vitreoretinal surgery is suggested to be the cause behind half of all sympathetic ophthalmia cases and, thus, up to 1 out of every 800 vitreoretinal cases [19].

Additionally, non-perforating injury has been documented to incite sympathetic ophthalmia, and as breakthroughs are made in ocular surgery and treatment, increased risk of sympathetic ophthalmia must be considered in clinical decision making. The use of laser cyclodestructive procedures for the treatment of glaucoma, particularly neodymium:YAG cyclodestruction has also been reported to cause sympathetic ophthalmia [57,58]. In addition to laser cyclotherapy, irradiation has been another non-perforating injury thought to be a risk for sympathetic ophthalmia. A 2012 retrospective study on 4,867 patients treated for choroidal melanoma with proton beam irradiation reported an incidence of 6.1 per 10,000 for sympathetic ophthalmia, finding that three patients developed sympathetic ophthalmia 3, 4, and 7 years following radiation treatment. The 0.06% incidence rate suggests that the incidence of sympathetic ophthalmia after proton beam irradiation may be low due to the ability of this treatment to target the tumor and spare adjacent normal tissue [59]. Sympathetic ophthalmia complicating helium ion irradiation of choroidal melanoma after 4 years [60] as well as following ruthenium plaque brachytherapy has been reported [61]. These findings indicate that incidence of the disease is rare following radiotherapy for uveal melanoma, but should still be acknowledged.

In addition to prevalence of the disease itself, recent study has taken to understanding incidence of ocular complications and vision loss associated with sympathetic ophthalmia. A study on 85 patients suggested that ocular complications were present in 47% of patients at the time of sympathetic ophthalmia presentation and that new complications occurred at an incidence of about 40% per year [62]. Additionally, increasing numbers of patients undergoing multiple surgeries may be additive in inciting sympathetic ophthalmia [63].

#### Developments in prevention, diagnosis, and therapies

While the classic prevention of sympathetic ophthalmia is maintained as enucleation of the injured eye before development of disease in the contralateral eye, controversy has long existed surrounding evisceration and whether it serves as an acceptable alternative to enucleation. Some studies suggest that the primary issue is the time-dependent removal of the eye; enucleation is cited as being the most successful for prevention of disease onset and worsening visual acuity if surgery is performed within 10 days post-injury. Others claim that early enucleation has no effect on visual outcome and, in fact, can have profound consequences since vision in the injured eye may ultimately be better than vision in the sympathetic eye [62].

While the debate between enucleation and evisceration was particularly robust following a controversial opinion published by Green et al. in 1972 [64], it appears that preference for evisceration over enucleation is currently increasing with advancements in technique and greater perceived benefits. In the past decade, only two cases of sympathetic ophthalmia following evisceration have been well documented; one case was not confirmed through histopathology, and the other is believed to have resulted from residual uveal tissue left in the sympathetic eye [65]. As there is substantial evidence of risk for sympathetic ophthalmia following any intraocular surgery, evisceration has the potential to be causative for sympathetic ophthalmia but has not been confirmed as a definitive risk for the disease. A retrospective study on patients from 1995 to 2004 showed that no cases of sympathetic ophthalmia were found in 491 primary eviscerations and 11 secondary eviscerations [66]. Taken with superior functional and cosmetic outcomes, evisceration appears to be favored in the most recent opinion. With improved outcomes and lowered complications of modern surgery, surgical repair of the injured eye may enable us to avoid enucleation and to confine or prevent antigen spreading that may initiate the development of sympathetic ophthalmia.

**Figure 3:**
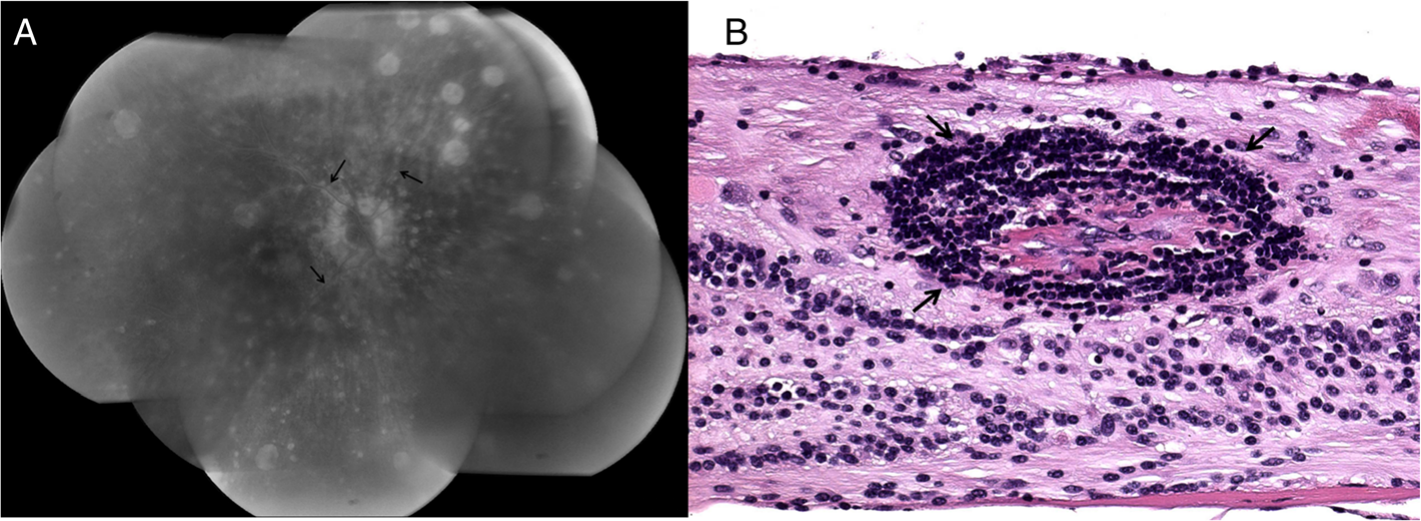
**Visualization of mild retinal vasculitis, an uncommon finding in sympathetic ophthalmia.** (**A**) Fluorescein angiogram of a sympathetic ophthalmia retina shows mild vascular leakage revealing retinal vasculitis (arrows). (**B**) Histopathology confirms retinal vasculitis with lymphocytes (arrows) alongside the vessel wall. Hematoxylin & eosin; original magnification, ×400.

The reliance on clinical findings for diagnosis has made ocular imaging a field of particularly high impact for sympathetic ophthalmia. Fundus photography, fluorescein angiography, and indocyanine green angiography have been useful in assessing ocular inflammation. Fundus photography can reveal yellow-white Dalen-Fuchs nodules and choroidal granulomatous infiltrates, vitreal cells and haze, retinal vasculitis, serous retinal detachment, and optic nerve swelling, as well as secondary glaucoma and cataracts [67]. Fluorescein angiography can show multiple hypo- and hyperfluorescing spots at the RPE level in the early venous phase, which then continues to late leakage (Figure 3). Dalen-Fuchs nodules can sometimes be visualized as early areas of hypofluorescence, and less commonly, retinal vasculitis can be observed in late staining (Figure 3) [18]; sometimes the optic nerve head may also stain [68]. Indocyanine green angiography can show areas of hypofluorescence where subretinal lesions are located, making it a useful adjunct to confirm diagnosis as well as a tool for monitoring response to treatment [67].

Recently, ocular coherence tomography (OCT) has been used to document retinal elevation in cases of sympathetic ophthalmia, allowing identification of serous retinal detachment and intraretinal edema, as well as revealing disintegration of RPE and disorganization of the inner retina. A 2006 study used serial OCT images to quantitatively monitor retinal status and demonstrate progression or improvement of serous detachment, suggesting OCT to be a reliable method of tracking response to treatment [69]. The extent of choroidal thickening by inflammatory infiltrate in sympathetic ophthalmia can be visualized through B-scan ultrasonography and can be useful in differentiating sympathetic ophthalmia from bilateral phacoanaphylactic endophthalmitis [67].

While systemic anti-inflammatory therapy has been the mainstay of sympathetic ophthalmia treatment for some time, the long-term use of corticosteroids has been called into question. Corticosteroid treatment is associated with cataracts and glaucoma, and systemic adverse effects such as diabetes mellitus, adrenal insufficiency, arterial hypertension, and osteoporosis must be considered carefully [70]. A report on corticosteroid-associated osteonecrosis in patients with chronic uveitis, although rare, advises minimization of systemic corticosteroid use when possible [71].

In cases refractory to corticosteroids and in patients with significant systemic side effects, immunosuppressive therapy that combines systemic corticosteroids and other immunosuppressive agents such as cyclosporine or azathioprine can improve prognosis, particularly in patients that have initial response to steroid but exhibit rebound activity when steroid is tapered to lower doses. The combination of corticosteroid with other different immunomodulators can be considered for cases that are refractory to steroid with a single immunosuppressive agent [41]. The use of mycophenolate mofetil or chlorambucil has also been shown to produce favorable outcomes in patients with refractory sympathetic ophthalmia [72,73].

Research into treatments that focus on mechanisms in the pathogenesis of sympathetic ophthalmia has produced a number of potential immune-related targets. Disruption of leukocyte recruitment by targeting gelatinase B (matrix metalloproteinase-9), CCL2, and CXCL12 may hold promise for future treatment [42]. Elevated CXCL11, CCL19, IL-18, and IL-23, and high IL-17 in sympathetic ophthalmia suggest targeting M1 macrophages and their cytokines and chemokines, Th17, or Th1 lymphocytes [46]. Targeting of oxidative stress-related molecules such as TNF-α in sympathetic ophthalmia may also be promising. Treatment with anti-TNF agents has been used for uveitis [74], suggesting potential benefit for sympathetic ophthalmia.

Alongside targeting of mechanisms within sympathetic ophthalmia pathogenesis, there have been pragmatic developments in disease management. A significant risk is undertaken with the administration of corticosteroids through intravitreal injection. One reported device that alleviates some of this risk is the flucinolone acetonide implant, providing continuous release of intraocular corticosteroid for approximately 2.5 years, reducing the need for systemic and local therapy [75]. Atan et al. found an association between cytokine gene polymorphisms and the severity of sympathetic ophthalmia, specifically that the IL-10 1082 SNP was associated with disease recurrence and level of steroid treatment required for management and that the GCC IL-10 promoter haplotype was protective against disease recurrence [76].

## Conclusions

While much has been gleaned about sympathetic ophthalmia since early reports in the nineteenth century, several challenges remain into the twenty-first century in efforts toward continuing research. The rarity of the condition has created a dearth of large-scale studies; to date, many reports on sympathetic ophthalmia are case studies of small numbers and are insufficient for a more widely applicable insight into mechanisms of action. Benefits of anti-inflammatory and immunomodulatory therapy in disease management are convincing, but significant variability in outcomes and adverse effects associated with the use of these therapies long-term suggest a future focus toward localized rather than systemic treatment and, importantly, a need for further studies into immunopathogenesis and novel targeted therapies for sympathetic ophthalmia.

## Competing interest

The authors declare that they have no competing interests.

## Authors' contribution

XKC wrote the manuscript. CCC provided critical review. Both authors read and approved the final manuscript.
